# Avian Feathers as Bioindicators of the Exposure to Heavy Metal Contamination of Food

**DOI:** 10.1007/s00128-013-1065-9

**Published:** 2013-08-04

**Authors:** Marcin Markowski, Adam Kaliński, Joanna Skwarska, Jarosław Wawrzyniak, Mirosława Bańbura, Janusz Markowski, Piotr Zieliński, Jerzy Bańbura

**Affiliations:** 1 Department of Experimental Zoology and Evolutionary Biology, Faculty of Biology and Environmental Protection, University of Łódź, Banacha 12/16, 90-237 Lodz, Poland; 2 Department of Teacher Training and Biological Diversity Studies, Faculty of Biology and Environmental Protection, University of Łódź, Banacha 1/3, 90-237 Lodz, Poland; 3 Natural History Museum, Faculty of Biology and Environmental Protection, University of Łódź, Kilińskiego 101, 90-011 Lodz, Poland; 4 Department of Ecology and Vertebrate Zoology, Faculty of Biology and Environmental Protection, University of Łódź, Banacha 12/16, 90-237 Lodz, Poland

**Keywords:** Nestlings, Feathers, Lead poisoning, Passerines

## Abstract

The aim of this study was to determine the possibility of using feathers of blue tit nestlings to assess the level of endogenous accumulation of lead. For this purpose we conducted an experiment with lead application to randomly chosen nestlings from eight randomly drawn broods. Five days after the exposure, feathers of lead-treated nestlings had significantly higher lead concentrations than control nestlings. This result suggests that feathers can be used as reliable non-destructive bioindicators to assess the level of heavy metals originating from contaminated food, which is of great significance for comparative studies on ecological consequences of pollution.

The natural environment has been gradually contaminated by various forms of pollution, mainly as a consequence of urbanization and the increasing use of fuels by households, vehicles and industry (Swaileh and Sansur 2006). A serious group of pollutants are heavy metals that pose a threat to all living organisms, with lead being especially considered as highly toxic (Roux and Marra 2007). The use of living organisms to monitor heavy metal pollution provides more promising results than chemical and physical analysis. This results from the fact that we obtain accurate data of bioavailability and biotransference of contaminants as well as observe some physiological and behavioral symptoms of induced toxicity (Swaileh and Sansur 2006; Roux and Marra 2007).

Birds can be exposed to heavy metals both externally, by physical contact, and internally, by consumption of contaminated food (Roux and Marra 2007). Therefore, nestlings of altricial birds, like blue tits *Cyanistes caeruleus*, seem to be an appropriate model for a study leading to discrimination between these two ways in which feathers can become contaminated (Furness 1993).

Due to the fact that the blue tit readily uses man-made nest-boxes, breeding populations can be easily established to monitor and study them in an area of interest, where it is possible to identify and evaluate local heavy metal pollution (Janssens et al. 2003).

Heavy metal concentrations can be assessed in birds by using various organs (liver, kidney), tissues (muscle, bone, fat), eggs, feathers and excrements (Burger 1993; Dauwe et al. 2000). An undisputable advantage of using feathers in such analyses is that they can be easily collected and, if necessary, repeatedly sampled without affecting the health and condition of studied individuals (Adout et al. 2007). During the period of growth, feathers are connected with blood-vessels and metals supplied with food may be built into feather keratine structures. Therefore, the endogenous accumulation reflects nestling physiological condition at the moment of feathering (Burger 1993). By contrast, exogenous contamination results from adsorption of heavy metals on the feather surface. Some authors also pointed out the fact that bird species, especially waterfowl and seabirds, may secrete metals through salt gland and embrocate them on their feathers (Dmowski 1999).

Birds were used to assess the amounts of lead especially in the aquatic environment, due to the widespread use of lead ammunition for hunting on waterfowl or lead weights used for fishing (Scheuhammer and Norris 1996). Studies on lead contamination in birds conducted in industrial areas showed a definite influence of environmental pollution on the levels of metals accumulated by birds (Dmowski 1993; Adout et al. 2007; Berglund et al. 2010), which is also true in urban areas (Janiga et al. 1990; Adout et al. 2007; Roux and Marra 2007). It should be emphasized that most studies were based on the trace element analyses of various tissues or internal organs, which inherently involved the necessity of sacrificing the studied individuals and only a few studies used exclusively feathers (Janiga et al. 1990; Adout et al. 2007).

The objectives of this study were to test whether increased levels of lead in the diet of blue tit nestlings would result in higher concentrations of this metal in their feathers and to determine if feather analysis could be used as a non-invasive method to monitor lead levels in the environment. In particular, we intended to demonstrate the applicability of this method in supporting state environmental monitoring programmes.

## Materials and Methods

The study was carried out in 2008 as a part of a long-term project on the breeding biology of tits around Łódź, central Poland. The study site was located in the central part of the Łagiewniki Forest (51°50′N, 19°29′E), situated in the northeast part of the city of Łódź. Tree stands of the site, covering c.140 ha, consist of mature deciduous trees of diverse species, with predomination of oaks *Quercus robur* and *Quercus petraea* (Bańbura et al. 2007). This area is characterized by reduced anthropogenic influence and limited car traffic.

During the 2008 breeding season, we conducted an experiment on 8 randomly drawn broods of blue tit. Regular inspections of nest-boxes allowed recording of the hatch dates of nestlings in order to set the proper day of conducting the experiment. In each brood, two randomly chosen nestlings (on the 10th day of their live) were administered through the gape, by using an automated pipette, 5 μL of lead acetate solution at a dose of 19 μg per gram of body weight. The lead acetate solution used was deliberately diluted (in deionized water) to a non-poisonous level (Eisler 1988). The remaining nestlings in each brood were treated as a control group, not receiving any dose. From both groups of nestlings, we collected secondary flight-feather samples on the 14th–15th day of their life. The feathers were kept at −18°C until further analysis. Although samples were stored and analysed in the laboratory individually for each nestling, the final comparison between experimentally treated and control nestlings was done on data pooled within these two categories of nestlings in particular broods.

Feathers taken to analysis were washed in deionized water to remove externally adsorbed heavy metals. Then they were dried for 24 h in the oven at 60°C until reaching constant dry mass and weighed to the nearest 0.001 g (Dauwe et al. 2000). Subsequently, the feathers were digested in a 4:1 mixture of 65 % nitrogenous acid and 70 % perchloric acid (Nyholm et al. 1995). Afterwards, all samples were diluted by adding deionised water up to 10 mL and were stored in polypropylene metal-free vials at −18°C until the time of analysis.

We measured lead concentrations in the samples using an atomic absorption spectrophotometer Spectr AA-300 with non-flame atomization in GTA96 graphite furnace tube atomizer. The limit of detection for lead was 0.02 ng/g. The analyses were conducted in the Laboratory of Computer and Analytic Techniques, Faculty of Biology and Environmental Protection, University of Łódź. All samples were analysed in batches, which included determination of calibration curves and use of blind samples. The values were expressed in  μg/g on a dry weight basis. Certified reference material (mussel tissue) from the Institute for Reference Materials and Measurements (Geel, Belgium) were used to ensure quality and accuracy of conducted analysis. The result for the certified reference material were within acceptable margins.

Statistical analyses of data were performed using STATISTICA 8.0. The unit observations were a per-brood mean lead concentration in feathers of treated nestlings and an analogous mean for control nestlings, resulting in obtaining a pair of data points for each brood. The data were ln-transformed in order to satisfy assumptions of normality and to stabilize variance. To test effects of the experimental treatment v. control treatment we used t-test for dependent samples (Sokal and Rohlf 1981) – the lead-acetate treated nestlings were compared with non-treated control nestlings from the same brood (n = 8 pairs of mean values). We assumed the level of significance at *p* < 0.05.

## Results and Discussion

**Fig. 1:**
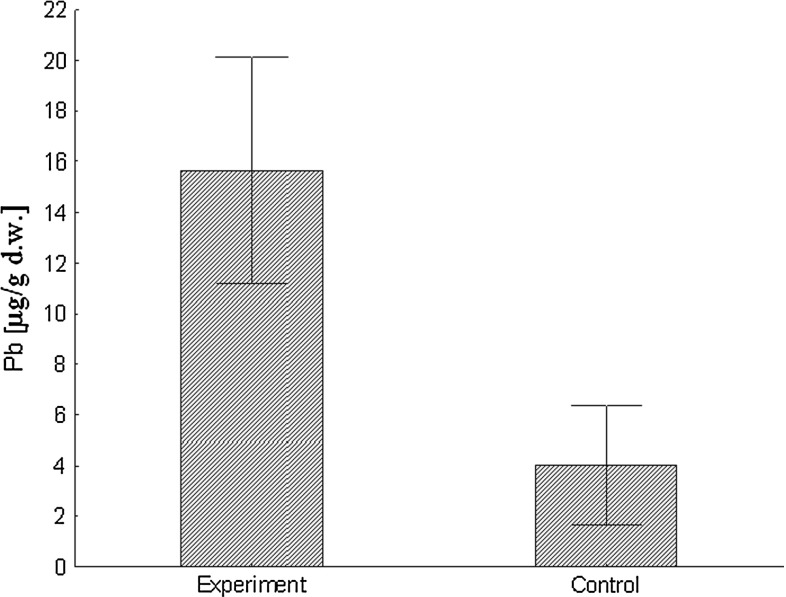
Concentration of lead (mean ± SD) in feathers of blue tit nestlings that were experimentally supplied with lead acetate in comparison with control nestlings

This study found a clear case of an endogenous pathway for lead deposition in feathers of nestling blue tits. Nestlings that were experimentally supplied with a solution of lead acetate contained higher lead concentrations in their feathers than control nestlings (paired *t* = 6.50, *df* = 7, *p* = 0.0003). Mean concentration of lead in feathers of lead acetate supplied nestlings was c. four times as high as the analogous mean concentration in control nestlings (Fig. 1). No detrimental effects on morphology, physiology or behavior were recorded in treated nestlings which resulted from the assumption that using such a dose of lead acetate solution would not contribute to toxicity.

These study findings provide an important result because it justifies the use of feathers as bioindicators of endogenous metal contamination that may originate from food. An alternative possibility was that feathers of nestlings would be contaminated by physical contact with externally contaminated parental birds. In our experimental system this result is especially valuable because Łódź urban agglomeration has been characterized as a contaminated area (Bem et al. 2003). For many years the pollution level in Łódź and its surroundings has been evaluated by conducting traditional physical and chemical analyses. Our study provides new data allowing investigators to consider the practical use of blue tit nestlings feathers to monitor the quality of environment in this part of Poland.

Experimental application of lead compounds to study the rate of lead accumulation in internal organs and feathers was documented by many authors. Experiments were conducted on farm birds such as ducks and quails (Kendall and Scanlon 1981a; Jeng et al. 1997) as well as on the wild-living bird species: ringed turtle-dove *Streptopelia risoria* (Kendall and Scanlon 1981b), starling *Sturnus vulgaris* (Dmowski 1993), mourning dove *Zenaida macroura* (Buerger et al. 1986), American kestrels *Falco sparverius* (Franson et al. 1983; Hoffman et al. 1985) and zebra finches *Taeniopygia guttata* (Dauwe et al. 2002).

Among these studies one can distinguish a group of experiments based on administering a single dose of lead. In the study conducted on zebra finches, Dauwe et al. (2002) showed that application of lead acetate solution at a concentration of 25 μg/mL resulted in significantly higher concentrations of lead in internal organs and feathers. The authors demonstrated that birds orally exposed to lead accumulated almost eight time higher values in their feathers than individuals from the control group. Buerger et al. (1986) in their study on the mourning dove *Z. macroura,* also showed that lead application causes significantly higher accumulation in liver and kidneys. In a study on American kestrel (Franson et al. 1983), after application of two lead doses (10 and 50 μg per gram of body weight) in food, the authors did not find significant differences in the concentration in liver between reference individuals (males and females) and those which received the lower dose of metal. Higher concentrations of lead were noted in liver of both genders as a result of the 50 μg/g dose. In another study, Kendall and Scanlon (1981a) conducted an experiment on the Japanese quail *Coturnix coturnix japonica.* They administered eight shot pellets to 10 adult males (one shot contained 74 mg of lead) and killed 10 birds using a pellet weapon. After 24 h, the concentration of lead was determined in chosen organs. There were no significant differences between lead concentration in bones and liver of reference birds and individuals killed by using the hunting weapon. However birds that received per os a dose of lead showed significantly higher levels of this metal both in bones and in liver.

Other studies show results of chronic lead administering. Conducting the experiment on starlings, in a strongly contaminated area (zinc smelter “Silesian Town” on Upper Silesia), Dmowski (1993) revealed that accumulation of lead in internal tissues clearly depends on the level of contaminants in food. After the application of metallic lead in three doses (25, 125, 625 μg per gram of body weight) to American kestrel nestlings from 1st to 10th day of their life, the concentration of this metal significantly increased (Hoffman et al. 1985). With increasing dose, Hoffman et al. (1985) noted enhancing level of lead in livers and kidneys. The highest dose induced 40 % mortality. In a different experiment, Jeng et al. (1997) supplied two 6-month-old mallard groups with the doses of lead compounds (10 and 20 μg per gram of body weight) every day for 3 months. Despite a lack of visible effects of lead toxicity (i.e. body mass reduction), a significantly higher lead concentration was found in analyzed tissues and organs in comparison to reference individuals. Similar results were shown by Kendall and Scanlon (1981b) in a study on ringed turtle-dove. The birds received 100 μg/mL of lead in drinking water during 2 weeks. This dose did not influence the reproductive cycle, however it caused increase in lead concentration in bones, liver and primary flight-feathers. Moreover, lead level in female bones was more than 10 times higher than in males.

## Conclusions

This study confirmed the hypothesis that analyzing metal content of feathers provides a reliable, non-invasive method of monitoring ecological consequences of the contamination of the environment. As representing structures of the body in which metal deposition occurs by the endogenous pathway, feathers can be as useful as internal organs in this respect. Consequently, they can be applied as bioindicators of metal pollution.
